# Do Patients with Psychosis See Their Symptoms the Same Way Clinicians Do?

**DOI:** 10.1192/j.eurpsy.2025.711

**Published:** 2025-08-26

**Authors:** P. Seif, G. Shashidhar, T. Khan, M. Keshavan

**Affiliations:** 1 Psychiatry and Mental Health, Beth Israel Deaconess Medical Center, Boston, United States

## Abstract

**Introduction:**

Psychosis includes positive (e.g., hallucinations) and negative symptoms (e.g., anhedonia), with tools like the PANSS traditionally used for evaluation. Although clinician-administered scales are considered the gold standard, patient self-reports provide critical insights into subjective experiences.

**Objectives:**

This study explores the discrepancies between patient-reported and clinician-assessed symptoms, aiming to improve psychosis diagnosis and treatment.

**Methods:**

Part of the BSNIP project, this study analyzed data from 159 participants (primarily male, average age 34.33) at the Boston site. Diagnoses were based on SCID, with most participants having schizophrenia, schizoaffective disorder, or bipolar disorder with psychotic features. The DSM-5 Level 1 Cross-Cutting Symptom Measure assessed psychiatric domains, including depression, anxiety, suicidal ideation, and psychosis, and was compared with clinician-reported assessments of the same symptoms.

**Results:**

Clinicians generally reported higher anxiety levels than patients with SZ (Z = -2.462, p = 0.014), while no significant differences were observed for the bipolar disorder (BP) and schizoaffective disorder (SAD) groups. Regarding suicidal ideation, patients typically reported higher levels than clinicians, particularly in the SZ group (Z = -3.507, p < 0.001) and the SAD group (Z = -2.007, p = 0.045). Similarly, patients in the BP (Z = -2.822, p = 0.005) and SAD (Z = -2.145, p = 0.032) groups reported more hallucinations compared to clinician assessments, while clinicians reported higher levels of hallucinations in the SZ group (Z = -3.451, p = 0.001). In terms of delusions, clinicians generally reported higher levels than patients in the SZ group (Z = -2.925, p = 0.003). Additionally, neither insight (PANSS_G12) nor cognitive function (BACS Composite) significantly impacted the discrepancies between patient and clinician reports of suicidal ideation, hallucinations, or delusions.

**Conclusions:**

The study highlights significant discrepancies in the reporting of anxiety, suicidal ideation, hallucinations, and delusions, especially in schizophrenia, where patients tend to underreport anxiety and psychotic symptoms but report higher suicidal ideation. Our findings point to the value of obtaining both patient and clinician assessments when evaluating psychosis.

**Disclosure of Interest:**

None Declared

